# The Structural Regulation and Properties of Energetic Materials: A Review

**DOI:** 10.3390/nano15151140

**Published:** 2025-07-23

**Authors:** Jin Yu, Siyu Xu, Weiqiang Pang, Hanyu Jiang, Zihao Zhang

**Affiliations:** National Key Laboratory of Energetic Materials, Xi’an Modern Chemistry Research Institute, Xi’an 710065, China; yujinnayo@163.com (J.Y.); jianghanyu613@126.com (H.J.); zzh565399203@stu.xjtu.edu.cn (Z.Z.)

**Keywords:** energetic materials, structure regulate, particle size, morphology, polycrystalline, core–shell, cocrystal, high-energy micro-units

## Abstract

Structural regulation is of great significance for improving the comprehensive performance of energetic materials (EMs). The structural regulation and properties of EMs were summarized. For single-component EMs, particle size control focuses on quality consistency and industrial scalability, morphology modification mainly improves sphericity through monomers or aggregates and explores the possibility of layered energetic materials in improving mechanical properties, and polycrystalline regulation suppresses metastable phases and explores novel crystalline forms using simulation-guided design. Composite EMs (CEMs) employ core–shell structures to balance safety with performance via advanced coating materials, cocrystal engineering to tailor energy release through intermolecular interactions, and lattice strain modulation, and mixing structures integrates component advantages while enhancing the reaction efficiency. Future directions emphasize computational simulations and novel fabrication methods to guide the rational design and precise preparation of next-generation EMs with specific functions.

## 1. Introduction

Energetic materials (EMs) play indispensable roles in rocket propulsion and safety devices, with extensive applications in aerospace, industrial, and civilian fields. With the advancement of modern technologies, the performance requirements for EMs have become increasingly stringent, demanding not only a higher energy density but also enhanced safety and stability standards [1,2,3,4]. Fundamentally, the properties of EMs are primarily determined by their chemical composition and molecular structure. Many researchers are dedicated to developing new energetic materials [5,6]. Meanwhile, this evolving demand has also driven the rapid development of structural regulation studies in EMs [7], aiming to achieve comprehensive performance improvements through the optimization of the microstructural architecture and chemical composition.

Structural regulation refers to the precise control of the molecular arrangement, crystal structure, morphology, particle size, and interface distribution via chemical or physical approaches. For single-component EMs, structural regulation primarily focuses on the particle size control [8,9], morphology modification [10,11,12], and polycrystalline structure [13]. For composite energetic materials (CEMs), it encompasses the design of core–shell structures [14], cocrystals [15,16], and mixing strategies [17,18], where synergistic interactions between different components contribute to performance enhancements. This multi-scale structural regulation methodology represents both a critical pathway for performance optimization and a theoretical foundation for developing novel EMs.

The remarkable progress in structural regulation research in recent years is exemplified by nanoscale engineering [19], which has enhanced energy release efficiency or cocrystal design strategies that balance high energy outputs with low sensitivity characteristics. However, significant challenges persist, including the insufficient precision in regulation techniques, an incomplete theoretical understanding of structure–property relationships, and scalability challenges in practical applications. Therefore, a systematic review and synthesis of current research achievements, coupled with an analysis of the correlations between structural regulation methods and performance optimization, holds substantial scientific significance and practical value.

Accordingly, this review systematically examines the recent progress in the structural regulation technologies for EMs, analyzes its role in performance improvement, and proposes future research trajectories, aiming to inform the further development of related fields.

## 2. Structural Regulation of Single-Component EMs

EMs serve as the foundational building blocks of modern energetic systems. The performance of EMs is primarily governed by intramolecular bonds and crystal structures, which dictate the critical balance between the energy density, thermal stability, and combustion behavior. Recent advancements in crystal growth methodologies, nanostructuring techniques, and polymorph-selective synthesis now provide robust strategies for tailoring material properties. These strategies operate through dimensional confinement, surface morphology regulation, and phase boundary engineering. The particle size reduction to nanoscale dimensions enhances surface-to-volume ratios, improving ignition sensitivity and energy release rates, while controlled morphological architectures—from needle-like crystals to spherical aggregates—directly influence the interfacial reactivity and mechanical robustness. Polymorphic transitions offer a pathway to achieve a higher energy density by stabilizing metastable, high-density crystal forms, while maintaining essential safety limits. This section examines these key structural regulation mechanisms and their multi-scale impact on energetic performance and functional reliability.

### 2.1. Particle Size and Particle Size Distribution

**Figure 1:**
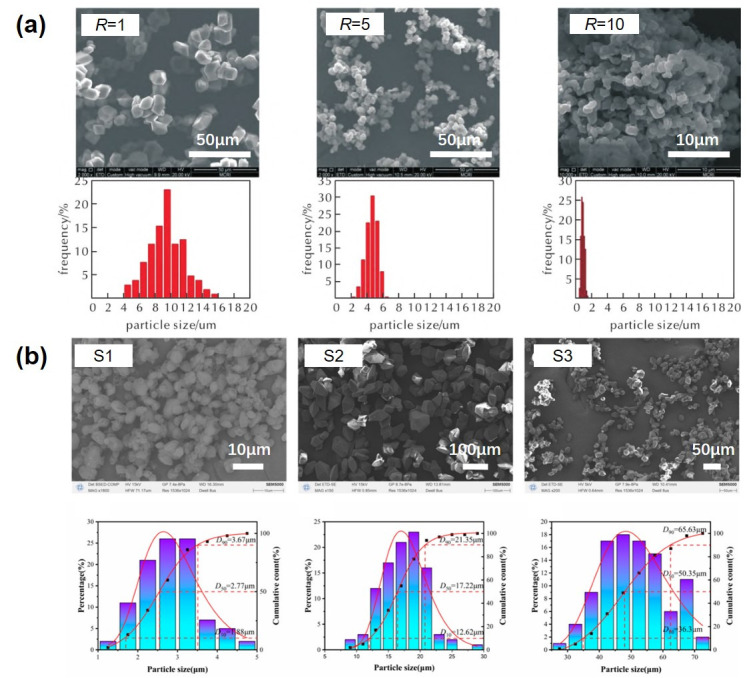
Morphology and particle size distribution images of HMX (**a**) [22] and CL-20 (**b**) [23].

Over the past few years, robust methodologies for particle size control across EMs have been established [20], with recent advancements increasingly focused on enhancing product consistency and process reproducibility. Microfluidic technology is widely employed for this purpose. For instance, microfluidic technology enabled the production of Cyclotrimethylenetrinitramine (RDX) with a median particle size (*D*_50_) of 3.35 μm, a SPAN number of 0.956 (SPAN is used to describe the width of particle size distribution. SPAN = (*D*_90_-*D*_10_)/*D*_50_. *D*_10_, *D*_50_, and *D*_90_ are the particle sizes corresponding to the cumulative particle size distribution of a sample reaching 10%, 50%, and 90%, respectively), and purity of 99.80% at a throughput of 207.7 g/h [21]. By optimizing flow dynamics, swirling chip structures suitable for the stable preparation of brittle crystals were identified. This approach facilitated the preparation of ultrafine Cyclotetramethylene tetranitramine (HMX, *D*_50_ = 8.86, 4.14, and 0.86 μm) and epsilon-Hexanitrohexaazaisowurtzitane (ε-CL-20, *D*_50_ = 2.77, 17.22, and 50.35 μm) [22,23]. The scanning electron microscope (SEM) images and the particle size distribution map obtained by the software are shown in Figure 1. The microfluidic system’s inherent advantages—including a small online sample quantity, high micro-mixing efficiency, and automation—enable the regulation and batch production of explosive crystallization at the safe critical scale.

There is also an innovative high-gravity-assisted solvent–antisolvent recrystallization method which has yielded sub-micron RDX particles (0.54 μm) [24]. However, compared with RDX, HMX (100 nm) demonstrated a superior detonation performance and reduced sensitivity. Specifically, its impact and shock sensitivities decreased by 107.0% and 62.1%, respectively, thereby making it a promising candidate for replacing raw RDX in composite modified double-base (CMDB) propellants [4,19]. In order to obtain HMX, a homogeneous emulsification method was developed to prepare HMX (1.38–3.40 μm) with a narrow particle size distribution and enhanced thermal stability (activation energy *E_a_* increased by 113.82 kJ·mol^−1^ and the critical temperature of thermal explosion increased by 6.23 K) [25]. Ultrasound-assisted crystallization has also improved the consistency, yielding uniform CL-20 microcrystals (*D*_50_ = 161 μm) while preventing agglomeration [26]. Despite these advances, challenges persist in scaling these sophisticated techniques while maintaining precise quality control and cost-effectiveness. Furthermore, translating laboratory-scale property improvements into reliable performance benefits in practical applications requires extensive validation.

The mechanistic understanding of particle size control has significantly advanced in recent years. Experimental and computational analyses now highlight the critical roles of supersaturation thresholds, solvent–crystal interactions, and diffusion dynamics in governing crystallization outcomes. For instance, Pal et al. [27] developed a predictive algorithm that correlates process parameters—the antisolvent pressure, temperature, stirrer speed, solution concentration, and nozzle diameter—with the resultant particle size. This model demonstrated a strong agreement with the experimental values, exhibiting a maximum absolute error of 11.52%.

### 2.2. Morphology

**Figure 2:**
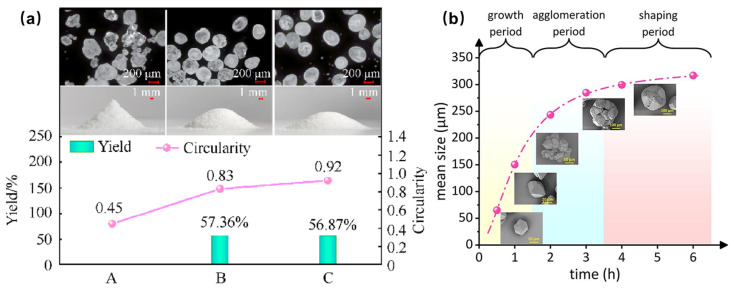
Micrographs and photographs of RDX (**a**) [30] and spherical evolution process of HMX (**b**) [31].

Spherical particles’ morphologies generally enhance the performance, including the flowability, sensitivity, and mechanical robustness [28,29]. The circularity of RDX prepared by the resonant sound mixing with the assisted solvent etching method can reach 0.92, and the *D*_50_ is 215.8 μm [30]. Micrographs and photographs of RDX are shown in Figure 2a. The thermal decomposition activation energy of spherical RDX was *E*_a_ = 444.68 kJ·mol^−1^, which is 47.76 kJ·mol^−1^ greater than that of raw RDX. Additionally, the minimum impact energy and frictional load were measured as 6.5 J and 144 N, respectively. HMX with a high purity (>99.5%), circularity (87.9%), and bulk density (1.17 g·cm^−3^) was also prepared through the condensation–dissolution mechanism [31]. In the experiment, the formation of spherical HMX includes three stages: a crystal growth period, an agglomeration period, and a shaping period (Figure 2b). The agglomeration period determines the size of the crystal, while the shaping period can significantly improve the circularity of the crystal.

**Figure 3:**
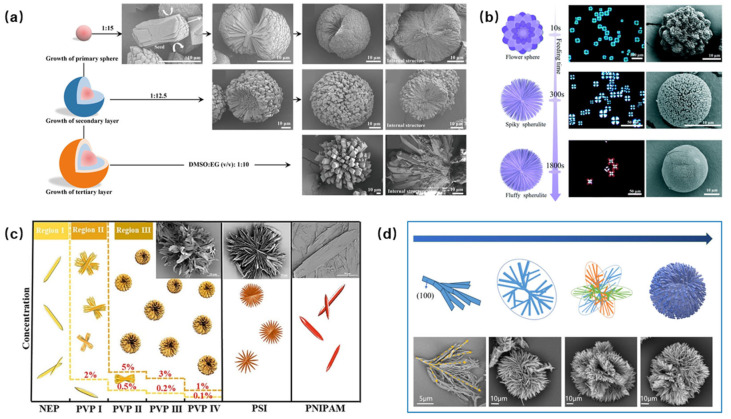
A schematic diagram of the morphology of different EMs. (**a**): The hierarchical evolution of HMX from single-crystallite into polycrystalline spherulites [32]. (**b**): The morphological tuning of the spherulitic aggregates with a different compactness [33]. (**c**): A schematic diagram of the morphology of HNS [34]. (**d**): A schematic diagram of the HNS spherulite growth process and SEM of the particle morphology at different growth stages [35].

Furthermore, some studies have gone beyond single-crystalline particles and constructed spherical polycrystalline aggregates with a radial non-uniform texture. Using polyvinylpyrrolidone as a growth additive and taking advantage of layer-by-layer crystallization, spherulitic HMX with different particle sizes can be obtained by adjusting the supersaturation (SEM images shown in Figure 3a). The radial crystal packing in these aggregates restricts the anisotropic expansion of subunits, inhibiting the β→δ phase transition and increasing its temperature by 20 K [32]. In addition, the morphology and the phase composition of the aggregates could be systematically tuned from δ-phase star-like aggregates to γ-phase flake spherulites by simply varying the HMX concentration (Figure 3b) [33]. Similarly, spherulitic, lamellar, and leaf-like 2,2′,4,4′,6,6′-hexanitroastragalus (HNS) crystals were prepared (Figure 3c) [34]. The spherulitic HNS exhibited nearly six times the specific surface area of the raw HNS, along with an improved impact insensitivity (40 J vs. 5 J) and friction insensitivity (12%, raw material: 4%). Combining the specific adsorption of toluene on the (100) crystal plane of HNS and the strong interaction between polyacrylic acid and each crystal plane of HNS, a compact HNS spherulite was prepared (Figure 3d) [35]. Due to the existence of internal pores, this spherical aggregate may have a more outstanding performance in increasing the combustion rate and uniformly loading the catalyst.

**Figure 4:**
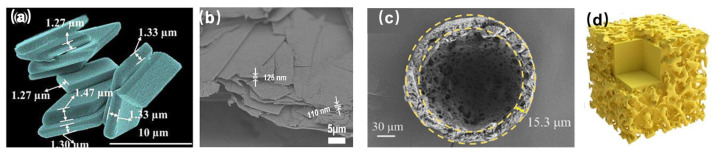
Images of RDX lamellae (**a**) [38], HMX lamellae (**b**) [37], porous hollow CL-20 (**c**) [39], and core@self-shell structure (**d**) [40].

Unconventional morphologies also enable performance tuning. Two-dimensional (2D) layered structures act as “energy converters,” dissipating mechanical stress through interlayer sliding and compression [36]. Both RDX lamellae (L–RDX, Figure 4a) and HMX lamellae (L–HMX, Figure 4b) were prepared [37,38]. The impact sensitivity of L–RDX is 4 J, and the friction sensitivity is 252 N, representing increases of 33.3 % and 16.7%, respectively, compared to raw RDX. The impact sensitivity of L–HMX is greater than 50 J, while the friction sensitivity is greater than 200 N. Meanwhile, L–HMX has a calculated velocity of detonation (VoD) of 9425 km·s^−1^ and a *P*_C-J_ of 41.5 GPa, which is even 5% higher than that of raw HMX. Hollow and porous hollow CL-20 microspheres (*D*_50_ = 506.8 μm, 318.6 μm, and 229.6 μm, Figure 4c) with a tunable shell thickness/pore size significantly intensified combustion flames and accelerated energy release [39]. Another type of core@self-shell structure, constructed by an original strategy of autologous surface molecular reconfiguration, with layered nano-surfaces (Figure 4d) was innovatively introduced into EMs, and the formation mechanism of “solvent micro-dissolved shells” was proposed [40]. The impact sensitivity (22.5 J vs. 12 J) has been significantly improved without significantly reducing the energy density. Such morphological innovations fundamentally redirect energy pathways—channeling external stimuli away from detonation initiation while preserving explosive power.

Computational tools are pivotal for morphology design. The crystal morphology prediction of ε-CL-20 in multiple solvents was achieved by using the improved adhesion energy model [41,42], and the influence mechanisms of the temperature, solvent type, and solvent composition on the crystal morphology were studied. However, the influence of experimental conditions on the crystal morphology cannot be ignored. The influence of crystallization parameters, such as the solvent system, solution concentration, crystallization temperature, additives, and stirring rate, on the crystal growth behavior of RDX has been studied [43]. It was found that with the gradual decrease in the supersaturation, the RDX crystal underwent a change process of rough growth–two-dimensional growth–helical growth, and the morphology of the RDX crystal gradually evolved from dendritic to octahedral. Therefore, it is a very important research direction for the simulation results to guide the experiments and for the experiments to verify and optimize the simulation details.

### 2.3. Polycrystalline

**Figure 5:**
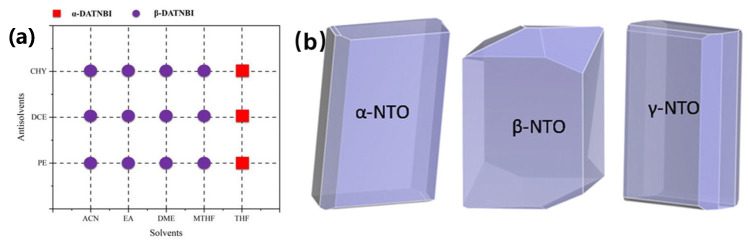
A schematic diagram of the crystal form of polycrystalline energetic materials. (**a**) The crystal forms of DATNBI in different solvent–non-solvent systems [49]. (**b**) Schematic diagrams of the three crystal forms of NTO [50].

Metastable phases dominate the initial crystallization process, yet typical nitroamine explosives exhibit distinct phase transition pathways. While RDX directly precipitates the thermodynamic α-phase through solvent–antisolvent recrystallization, HMX and CL-20 follow the Ostwald step rule due to the high activation barrier for stable nucleation [12,44,45]. The microfluidic technology enables the precise control of the polymorphisms of CL-20 and HMX [46,47]. The metastable phases β-CL-20 (*D*_50_ = 1.04 μm) and γ-HMX were formed preferably and transformed into ε-CL-20 (*D*_50_ = 50.35 μm) and β-HMX after maturation. Although stirring accelerates the transformation, it compromises crystal perfection, revealing the trade-off between kinetic efficiency and thermodynamic control. Solvent-mediated phase transitions have emerged as a new strategy for obtaining energy-beneficial but kinetically inaccessible polycrystalline forms. At the molecular level, density functional theory tight binding molecular dynamics (DFTB-MD) and density functional theory (DFT) have revealed the solvent-specific mechanisms controlling polymorphic stability [48]. In the saturated solution of tetrahydrofuran, the solute clusters of 4,4′,5,5′-tetranitro-1H,1′H-[2,2′-biimidazole]-1,1′-diamine (DATNBI) preferentially form solvated monomers. The solvate composed of DATNBI and tetrahydrofuran (THF) can be easily transformed into the scarce α form of DATNBI after desolvation (Figure 5a) [49]. A new crystal form of γ-3-nitro-1,2,4-triazole-5-one (γ-NTO, space group *P*c, density of 1.907 g·cm^−3^, the schematic diagram of the crystal form is shown as Figure 5b) was also developed through solvent evaporation [50], combining an excellent detonation performance with structural symmetry.

Based on the above, the research related to the structural regulation and properties of single-component EMs is summarized in Table 1. Single-component EMs leverage structural engineering to balance energy density, stability, and reactivity. Particle size regulation prioritizes nanoscale precision to enhance ignition sensitivity and energy release rates, while ensuring batch-to-batch consistency for industrial scalability. Morphology control focuses on improving sphericity through tailored crystallization (e.g., spherical aggregates) for superior flowability and impact resistance, alongside exploring layered structures to strengthen mechanical robustness. Polymorphic engineering targets stabilizing high-energy phases via the kinetic suppression of metastable forms, complemented by computational simulations to predict novel thermodynamically favorable crystal structures. These strategies collectively address the critical trade-off between energetic efficiency and safety, guiding the design of advanced EMs with controlled defect distributions and optimized interfacial interactions.

## 3. Structural Regulation of CEMs

CEMs demonstrate performance advantages that single-component materials cannot achieve through multi-component synergistic design and multi-scale structural regulation. These materials are centered on high-energy compounds, incorporate binders, oxidizers, fuels, or functional additives to construct specific spatial configurations via advanced materials science methodologies, thereby achieving an organic integration of energy density, safety performance, and functional properties. The current structural regulation of CEMs primarily focuses on four typical modes: core–shell (coating) [51], cocrystal [52,53], and mixing [54]. In recent years—driven by rapid advancements in nanotechnology, supramolecular chemistry, and micro/nanofabrication techniques—the structural regulation of CEMs has evolved into a comprehensive system spanning from molecular-level assembly to macroscopic functional integration [55], exhibiting revolutionary application prospects in explosives, propellants, and pyrotechnics.

### 3.1. Core–Shell

**Figure 6:**
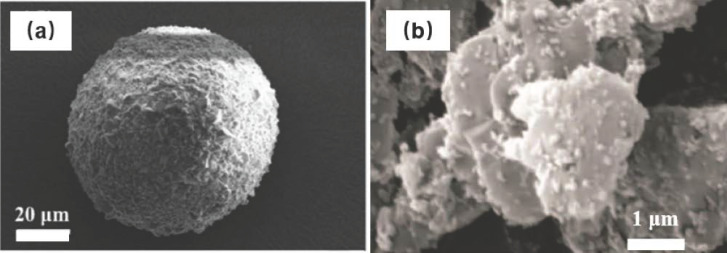
Morphology images of CL-20@LLM-105 prepared by Pickling emulsion method (**a**) [59] and microfluidic method (**b**) [60].

The core–shell structure mainly utilizes the protective and functional properties of shell components to balance the limitations of EMs cores, enhancing safety performance [56]. For example, the CL-20@collodion (NC) microspheres (0.5–5 μm) are prepared via spray drying, which exhibit an 18.65 kJ·mol^−1^ increase in *E*_a_ and a threefold increase in the impact sensitivity of the raw CL-20 [51]. Similarly, a three-layer structure of a CL-20/Al@C@polybutadiene (BR)/wax powder composite retains the *β*-CL-20 crystal form while demonstrating reduced sensitivities, with friction and impact sensitivity, as measured by explosion probabilities of 36% and 38%, respectively, and the characteristic drop height of the impact sensitivity is 13.4 cm. This composite maintains a detonation heat of 5946 J·g^−1^, which is 97.91% of the raw CL-20 (6073 J·g^−1^), limiting the energy loss to under 3% [57]. Further desensitization was achieved using an interfacial crosslinking anti-falling composite wax-film on CL-20 [58], yielding an impact sensitivity superior to a polymer-bonded explosive (PBX) containing 95% 2,6-diamino-3,5-dinitropyrazine-1-oxide (LLM-105) and 5% ternary composite. This coating increased the *E*_a_ by over 375.27 kJ·mol^−1^, the peak temperature (*T*_p0_) by 21.04 K, and the critical temperature (*T*_b_) by 18.27 K. Coatings with insensitive explosives can further ensure the overall energy performance of the composite materials. The Pickling emulsion method and microfluidic method were used to prepare CL-20@LLM-105 composites with particle sizes of 50–100 μm and 0.19–3.92 μm, respectively [59,60]. The microscopic morphology of CL-20@LLM-105 is shown in Figure 6. The former showed a greater roundness and stability (thermal decomposition temperature was 9.4 K higher than raw CL-20, *H*_50_ was 13 cm higher, and explosion probability was 43% lower).

**Figure 7:**
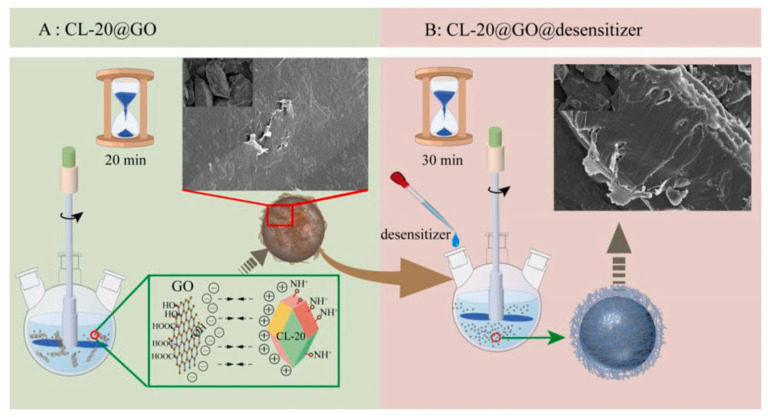
The flow chart of the composite particles preparation process [63].

Carbon-based materials offer distinct advantages, including a high thermal conductivity, lubrication, and functionalized flexibility. For instance, a graphene oxide (GO) coating elevated the activation energy of CL-20 by 63.0 kJ·mol^−1^, improved the *H*_50_ from 13.0 cm to 23.5 cm, and reduced the friction sensitivity to 24% [61]. Carbon nanotube (CNT) coatings increased the elastic modulus and hardness of HMX by 303% and 311%, respectively, while reducing the impact and friction sensitivity by 68.5% and 233% [62]. Furthermore, multi-coating materials such as the CL-20@GO@desensitizer composite (Figure 7) [63] and the HMX-polyethyleneimine@reduced GO/g-C_3_N_4_ (HPrGC) composite [64] were innovatively prepared based on the enhancement of the interface effect of GO. The CL-20@GO@ethylene propylene diene monomer (EPDM) composite exhibited an *E*_a_ of up to 351.43 kJ·mol^−1^, whereas the *E*_a_ of CL-20@GO was merely 184.36 kJ·mol^−1^_._ The perfect combination of g-C_3_N_4_ and rGO endows HMX-PEI@rGO/g-C_3_N_4_ with an ideal mechanical sensitivity (IS: 21 J, FS: 216 N). For these multi-coated composite materials, the shell sequence will also have an impact on the overall effect [65].

Biomimetic polymers have emerged as effective shell materials. The microdroplet self-assembly platform combined with polydopamine (PDA) in situ encapsulation technology prepared HMX/fluoropolymer (F2604)@PDA microspheres with a controllable shell thickness [66]. The 4.96 μm PDA shell delayed the thermal decomposition by 9.67 K, reduced the mechanical sensitivity by 22%, and improved the powder flowability. In addition, the PDA shell has a significant impact on the combustion performance. An interface modification using tea polyphenols (TPs) increased the RDX thermal decomposition temperature by 4.4 K [67]. The enhanced interaction between TiO_2_ nanoparticles and CL-20 by hydrogen bonding resulted in an increased *T*_p_ of 9.79 K and 9.09 K for CL-20@TP-TiO_2_ and CL-20@DA-TiO_2_, respectively, compared to raw CL-20 [68].

Function-targeted core–shell structures address specific challenges. Ammonium dinitramide (ADN)@HMX increased the water contact angle to 98.92°, reduced the hygroscopicity by 80%, and enabled stable burning for 6 h at 68% relative humidity [69]. Introducing the conductive polymer Poly(3,4-ethylenedioxythiophene):poly(styrenesulfonate) (PEDOT:PSS) into RDX reduced the electrostatic spark sensitivity by 40% [70].

Recrystallization-based structural regulation must address crystal form stability issues in metastable materials like HMX and CL-20. For instance, *ε*-CL-20 in propellant systems can undergo a crystal form transformation upon contact with additives, resulting in the volume expansion of the entire propellant system and the formation of cracks. This was mitigated by constructing polyphenolamine (PCHA) films to block the solvent contact, increasing the crystal form transition temperature by 16 K and the *T*_p_ by 7 K [71]. *β*-HMX@PDA particles obtained through supersaturation control exhibited a friction and impact sensitivity of 36% and 56%, respectively, representing 52% and 28% reductions compared to raw HMX [72].

The implementation of core–shell structures enhances the safety, stability, and functionality of EMs by leveraging the protective and functional advantages of shell materials. Crucially, the introduction of non-energetic shell materials inevitably entails a trade-off in energy density. The precise control of the shell content is paramount to effectively balance this inherent energy loss against significant performance gains in sensitivity reductions, thermal stability, and processability. This optimization represents a critical research focus. Furthermore, the diversity of available shell materials enables tailored functionalities. Looking ahead, the exploration of novel shell materials remains highly promising.

### 3.2. Cocrystal

**Figure 8:**
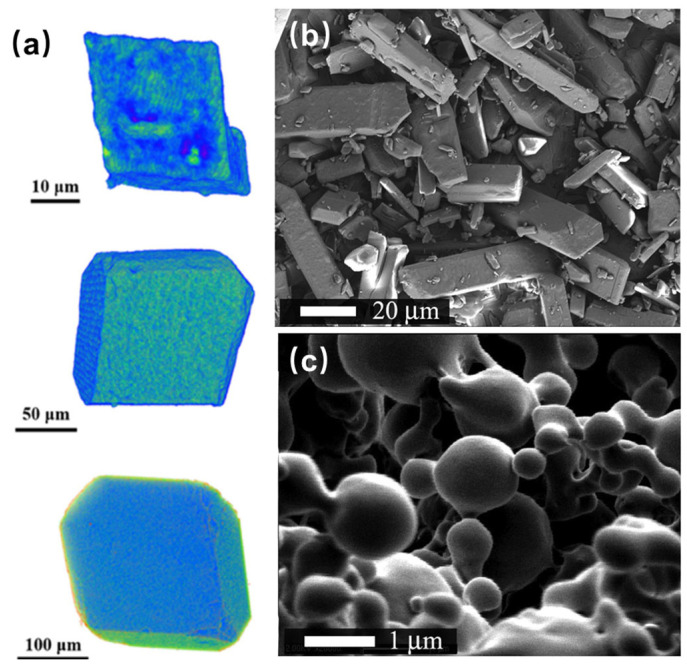
(**a**) Three-dimensional reconstructions and profile analysis of CL-20/HMX cocrystal [75]. (**b**,**c**) SEM images of CL-20/MTNP cocrystal preparation by solvent evaporation method and spray drying method [78].

Cocrystal engineering enables the precise regulation of energetic materials by tailoring intermolecular interactions, thereby enhancing the energetic density, thermal stability, and combustion efficiency. This strategy balances safety and performance through controlled crystal packing, offering tunable properties for advanced EMs. The rigid cage molecular structure and six flexible NO_2_ groups of CL-20 facilitate cocrystal formation, exemplified by cocrystals with HMX that exhibits a reduced sensitivity [73,74]. Three different rhombohedral CL-20/HMX cocrystal samples with a uniform particle size distribution (*D*_50_ = 20, 63 and 130 μm) were prepared. A micro-computed tomography analysis indicates that large-grain samples have fewer internal defects and better crystal integrity (Figure 8a) [75]. Spray drying demonstrates advantages for the preparation of cocrystals by facilitating the kinetic entrapment of thermodynamic instable cocrystals [76]. Based on this, the rapid preparation of ultrafine CL-20/2,4,6-trinitrotoluene (TNT) cocrystals has been achieved, which solves the problems of slow crystallization rates, large particle size variations, complex process conditions, and the low safety of cocrystal explosives [77]. Spherical CL-20/1-methyl-3,4,5-trinitropyrazole (MTNP) cocrystals (0.2–2 μm, Figure 8b,c) have been prepared via spray drying, achieving a slapper impact threshold lower than HNS [78]. A novel CL-20/FOX-7 cocrystal (molar ratio of 1:1) demonstrates better thermal stability compared to CL-20/TNT cocrystals and exhibits a lower mechanical sensitivity than both CL-20/TNT and CL-20/HMX cocrystals. Its crystal density and detonation parameters are much higher than CL-20/TNT and are slightly lower than CL-20/HMX [79]. An ADN/CL-20 cocrystal with improved hygroscopicity was designed and prepared [80]. The hydrogen bonds formed between ADN and CL-20 saturate the ammonium ions of ADN, inhibiting moisture absorption. This cocrystal achieves a high specific impulse (*I*_sp_ = 272.6 s). The HMX/1,1-diamino-2,2-dinitroethylene(FOX-7) cocrystal can be stably obtained under low-temperature conditions [81]. By adjusting the molar ratio, the formation of either FOX-7/HMX cocrystals or physical mixtures can be controlled [82], which proves the multi-functionality and morphological regulation enabled by hydrogen bonds.

**Figure 9:**
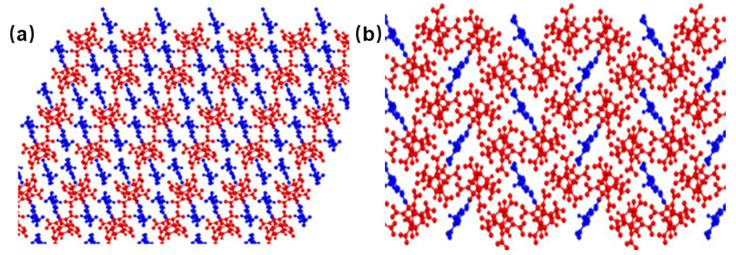
Crystal structure of 1:2 CL-20/3,4-MDNP (**a**) and 2:1 CL-20/3,5-MDNP (**b**) [84].

Theoretical research is accelerating the high-throughput screening and performance prediction of cocrystal structures. A graph neural network (GNN)-based deep learning framework has been pioneered to predict the cocrystal formation, accelerating material discovery [83]. Simple and effective methods based on the structural similarity and hydrogen bond pair energy have also been used for the screening of CL-20 cocrystals. Combined with the experimental research, two new CL-20 cocrystals were successfully obtained, namely 1:2 CL-20/3,4-MDNP(1-methyl-3,4-dinitropyrazole) and 2:1 CL-20/3,5-MDNP(1-methyl-3,5-dinitropyrazole) (Figure 9) [84]. Notably, 2:1 CL-20/3,5-MDNP demonstrates a high density (1.889 g·cm^–3^), excellent detonation performances (9079 m·s^–1^, 37.30 GPa), and low sensitivity (12 J). Through simulation calculations, the crystal structure, electronic properties, optical properties, mechanical properties, and Hirshfeld surface interactions of the CL-20/4, 5-MDNI (1-methyl-4,5-dinitroimidazole) cocrystal with two different stoichiometric ratios under high-pressure conditions were also studied [85], revealing the stoichiometric-dependent structure and mechanical properties.

### 3.3. Mixing

**Figure 10:**
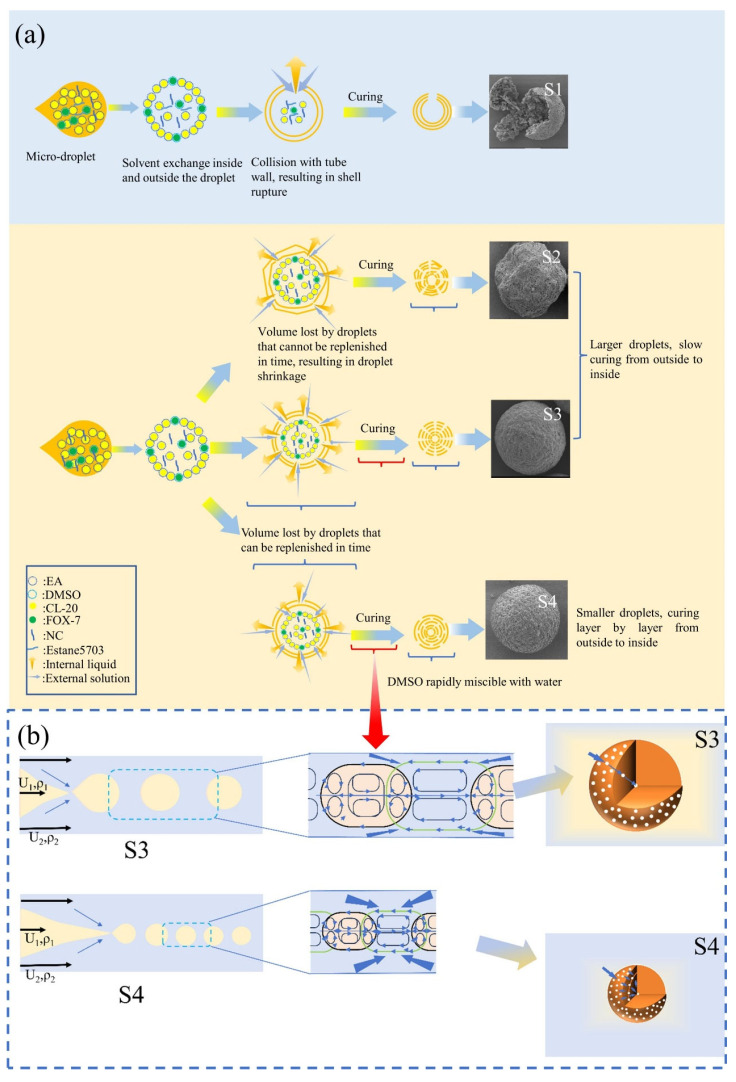
Formation process of CL-20/FOX-7 composite microspheres with hollow and porous structures [91]. (**a**) Hollow structure CL-20/FOX-7. (**b**) Solide structure CL-20/FOX-7.

One or more materials are assembled through binders to form mixing structure composite materials of different sizes and shapes. Commonly used binders include glycidyl azide polymer (GAP), NC, fluororubber, etc. Nitroglycerin ether cellulose (NGEC), a type of high-energy binder, has been innovatively used in the assembly of RDX [86]. The mechanical properties in terms of the impact strength of NGEC/RDX-GPs with different contents of NGEC was improved by 15.3%~117.1%, 3.9%~34.6%, and 6.9%~31.1% under conditions of −40 °C, 20 °C, and 50 °C; the compression strength was improved by 2.5%~23.1%, 10.7%~27.9%, and 7.3%~28.5%, while the tensile strength was improved by 15.4%~35.0%, 10.4%~33.0%, and 11.8%~35.5%, respectively. Most related studies have adopted microdroplet technology and the Pickering emulsion method. Composite materials such as HNS/CL-20 [87], 1,1′-dihydroxy-5,5′-bitetrazolyl dihydroxyamine salt (TKX-50)/CL-20 [88], and FOX-7/HMX [17] have been reported. Both methods were applied for the preparation of TATB(2,4,6-trinitrobenzene-1,3,5-triamine)/HMX composites [89,90]. The microdroplet method achieves morphological regulation (erythroid and spherical). The morphology of the composite material can be controlled by adjusting the content of the binder. For instance, in the CL-20/FOX-7 composite, the low content of the binder results in fragile shells that rupture during transportation due to the tube wall friction, forming hollow structures (Figure 10a, S1) [91]. Increasing the binder content delays crystal precipitation, preventing the compensation for the volume loss by solvent exchange and leading to porous irregular microspheres. The droplet size significantly influences solidification kinetics. Faster-flowing dispersed phases produce larger droplets (Figure 10a,b, S2/S3), where an insufficient continuous-phase circulation causes delayed inward solvent exchange through microporous channels and heterogeneous precipitation. In contrast, a slower flow generates smaller droplets (Figure 10a,b, S4) with an efficient solvent exchange, maintaining a high-phase purity. This enables sequential shell solidification, where the newly formed water phase penetrates shell pores to replicate outer structures through layer-by-layer co-precipitation, ultimately forming uniform core–shell microspheres. The key mechanism involves a dynamic balance between the binder concentration, crystal nucleation kinetics, and solvent diffusion rates.

**Figure 11:**
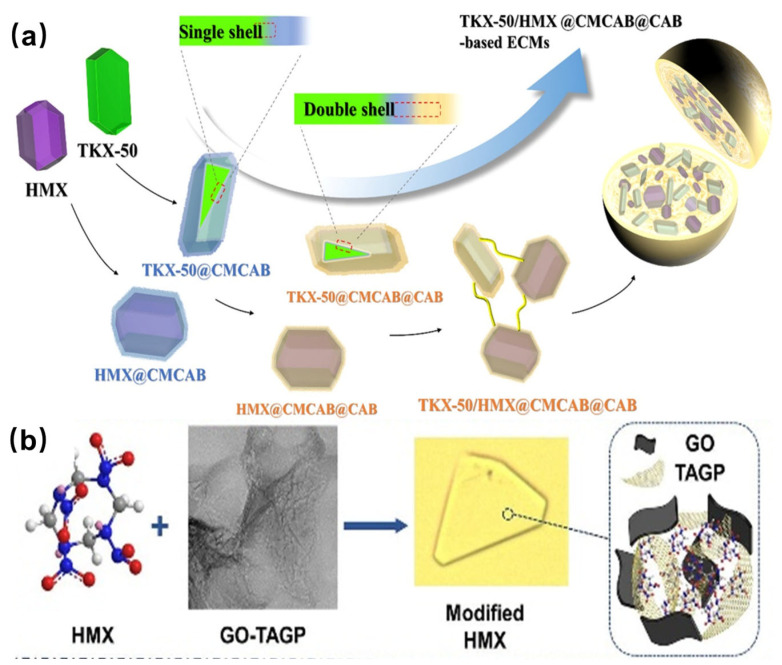
Schematic diagram of core@double-shell structured (**a**) [92] and GO-TAGP intercalated HMX crystals (**b**) [93].

Additionally, specialized mixing structures have been developed. A carboxymethyl cellulose acetate butyrate@cellulose and acetate butyrate (CMCAB@CAB) two-layer binder system (Figure 11a) addressed the poor interfacial compatibility of TKX-50, achieving stable molding into a smooth column and reducing sensitivity (frictional load up to 324 N) [92]. Physical cross-linked polymers spontaneously assembled with κ-carrageenan (KC) and chitosan (CTS) exhibit a dual-action mechanism, synergistically mitigating the corrosive properties of NTO and enhancing its insensitivity threshold [18]. Compared to NTO, the NTO/KC/CTS exhibited a 14.4 K decrease in the thermal decomposition temperature and a substantial reduction in the *E*_a_ by 290.9 kJ·mol^−1^. HMX crystals with an increased energy density (measured VoD: 9254 m·s^−1^; impact energy: 26.5 J) but decreased sensitivity (friction sensitivity: >360 N) were prepared by embedding triamine–guanidine–glyoxal cross-linked graphene oxide (GO-TAGP), which induced a transformation of the HMX crystal structure to the γ-type (Figure 11b) [93]. Luo et al. [94] developed a binder-free method for preparing spherical TKX-50/TATB composites. Xue et al. [95,96] integrated nanoscale insensitive additives (TATB@PDA, FOX-7@PDA, LLM-105@PDA) into the CL-20 matrix to form football-shaped or walnut-shaped co-particles [97] with dense CL-20 shells, highlighting the role of interfacial hydrogen bonds and electron-withdrawing interactions.

**Figure 12:**
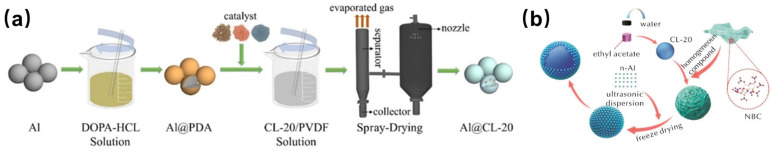
A schematic diagram of the preparation process of Al@CL-20/PDA (**a**) [99] and Al/CL-20@NBC (**b**) [105].

An important application of the mixing structure is the regulation of high-energy micro-units (EMUs). EMUs are defined as nanoscale precise composite materials of oxidants and fuels with customized oxygen-to-fuel ratios. It integrates the advantages of the microstructure design and surface modification of Al to improve the energy release characteristics of Al in mixing explosives. The preparation process involves first coating the fuel, then constructing a three-dimensional network through binders to assemble it with oxygen to form uniformly distributed composite materials (Figure 12a). This structure effectively shortens the interfacial heat and mass transfer distance for the fuel. Meanwhile, oxidant decomposition generates gaseous substances that disperse the fuel particles, which is conducive to reducing the agglomeration effect. Based on this strategy, EMUs such as CL-20/Al@Co/nitrifying bacteria cellulose (NBC) [98], Al@CL-20/PDA (Figure 12a) [99], Al@HMX/PDA [100], HMX/B/Al/PTFE [101], hollow CL-20/Al/F2605 [102], and TKX-50/Al/GAP [103] have been fabricated. The particle size of RDX/Al/NC [104]. Experimental results were slightly smaller than simulations but followed the same trend: a higher Al content resulted in a larger particle size. Using explosives as oxidants may pose safety concerns. Some studies have avoided this by forming a metal fuel shell layer [105,106,107,108]. Its preparation method and principle remain unchanged (Figure 12b). The performance enhancement of EMUs is mainly due to the synergistic effect among components and the advantages of the core–shell morphology and structure, making full use of the energy balance strategy of high-energy components and the structural regulation of component interfaces to improve the combustion performance and energetic performance of composite materials while reducing the sensitivity [54]. The rheological properties, mechanical performance and pressure index of EMUs meet the loading requirements of solid propellants [109], demonstrating a significant application potential.

Based on the above, the research related to the structural regulation and properties of CEMs is summarized in Table 2. Core–shell structures primarily enhance safety by encapsulating sensitive high-energy compounds within protective shells, with recent efforts focusing on multi-functional coatings to reconcile safety–performance trade-offs. A cocrystal enables tailored energy release profiles through controlled intermolecular interactions, optimizing the detonation efficiency while mitigating sensitivity via the lattice strain modulation. Mixing strategies exploit multi-component dispersion at micro/nanoscales—e.g., embedding oxidizers/fuels in polymer matrices or forming heterogeneously distributed energetic micro-units—to synergize individual properties and accelerate reaction kinetics through improved interfacial connectivity. These approaches collectively address the critical challenge of balancing the energy output, stability, and processability in CEMs, driven by advances in computational design and precision fabrication techniques like 3D printing or microfluidics.

## 4. Conclusions

Structural regulation has emerged as a pivotal strategy to optimize the performance of EMs while balancing the energy output and safety performance. For single-component EMs, precise control over the particle size enhances the combustion efficiency and reduces sensitivity, whereas tailored morphologies improve the stability and processability. Polymorphic transitions further enable property modulation by exploiting phase-dependent energetic and mechanical traits. In CEMs, core–shell architectures mitigate reactivity risks by isolating sensitive components, cocrystallization achieves a homogeneous energy distribution and enhanced detonation performance, and mixing composites offer tunable property trade-offs through multi-scale structural integration. These approaches collectively address critical challenges in the energy density, thermal stability, and impact sensitivity of EMs.

In the future, priority should be given to conducting more in-depth theoretical research and developing correlation models between structural characteristics and dynamic behaviors and to establishing a safer and mass-production-capable preparation technology to achieve the precise regulation of EMs with microscopic, mesoscopic, and macroscopic structures. The importance of the practical application and further exploration of the potential of structural regulation in chemical propulsion will be stressed.

## Figures and Tables

**Table 1:** Structural regulation and properties of single-component EMs.

Structural Regulation	Materials	Properties	Reference
Particle size and particle size distribution	RDX	*D*_50_ = 3.35 μm, SPAN = 0.956, and purity = 99.80%	[21]
		0.54 μm	[23]
	HMX	*D*_50_ = 8.86, 4.14 and 0.86 μm	[22]
		100 nm, impact and shock sensitivities decreased by 107.0% and 62.1%	[19]
		1.38–3.40 μm, *E_a_* increased by 113.82 kJ·mol^−1^, and the critical temperature of thermal explosion increased by 6.23 K	[25]
	CL-20	*D*_50_ = 161 μm	[26]
		*D*_50_ = 2.77, 17.22 and 50.35 μm	[23]
Morphology	RDX	Circularity = 0.92, *D*_50_ = 215.8 μm, *E*_a_ = 444.68 kJ·mol^−1^, impact sensitivity is 6.5 J, and friction sensitivity is 144 N	[30]
		Lamellae, impact sensitivity is 4 J, friction sensitivity is 252 N	[38]
	HMX	Circularity = 0.92, bulk density = 1.17 g·cm^−3^	[31]
		Spherulite aggregates, the transformation temperature increasing by 20 K	[32]
		Lamellae, impact sensitivity > 50 J, friction sensitivities > 200 N, VoD = 9425 km·s^−1^, *P*_C-J_ = 41.5 GPa	[37]
	CL-20	Hollow and porous hollow, *D*_50_ = 506.8 μm, 318.6 μm, and 229.6 μm	[39]
	HNS	Spherulite aggregates, impact sensitivity is 40 J (raw material: 5 J), and friction sensitivity is 12% (raw material: 4%)	[35]
Polycrystalline	CL-20	β-CL-20 (*D*_50_ = 1.04 μm), ε-CL-20 (*D*_50_ = 50.35 μm)	[46]
	HMX	γ-HMX, β-HMX	[47]
	NTO	γ-NTO	[50]
	DATNBI	α-DATNBI, β-DATNBI	[49]

**Table 2:** Structural regulation and properties of CEMs.

Structural Regulation	Materials	Properties	Reference
core–shell	CL-20@NC	0.5–5 μm, *E*_a_ increased by 18.65 kJ·mol^−1^, impact sensitivity increased by 3 times	[51]
	CL-20/Al@C@BR/wax	friction sensitivity is 36%, impact sensitivity is 13.4 cm, heat of explosion is 5946 J·g^−1^	[57]
	CL-20@composite wax-film	*E*_a_, *T*_p0_, and *T*_b_ increased by >375.27 kJ·mol^−1^, 21.04 K, and 18.27 K, respectively	[58]
	CL-20@LLM-105	50–100 μm and 0.19–3.92 μm, *H*_50_ increased by 13 cm	[59,60]
	CL-20@GO	*E*_a_ increased by 63.0 kJ·mol^−1^, impact sensitivity is 23.5 cm, friction sensitivity is 24%	[61]
	HMX@CNTs	elastic modulus and hardness increased by 303% and 311%, impact and friction sensitivity reduced by 68.5% and 233%	[62]
	CL-20@GO@EPDM	*E*_a_ = 351.43 kJ·mol^−1^	[63]
	HMX-PEI@rGO/g-C_3_N_4_	impact sensitivity is 21 J, friction sensitivity is 216 N	[64]
	HMX/F2604@PDA	*T*_p_ increased by 9.67 K	[66]
	CL-20@TP-TiO_2_	*T*_p_ increased by 9.79 K	[68]
	CL-20@DA-TiO_2_	*T*_p_ increased by 9.09 K	[68]
	RDX@TP	*T*_p_ increased by 4.4 K	[67]
	ADN@HMX	water contact angle is 98.92°, still burns stably for 6 h at 68% humidity	[69]
	RDX@ PEDOT:PSS	electrostatic spark sensitivity reduced by 40%	[70]
	CL-20@ PCHA	*T*_p_ increased by 7 K, crystal transition temperature increased by 16 K	[71]
	*β*-HMX@PDA	friction sensitivity is 36%, impact sensitivity is 56%	[72]
cocrystal	CL-20/HMX	*D*_50_ = 20, 63 and 130 μm	[75]
	CL-20/TNT	<10 μm	[77]
	CL-20/MTNP	0.2–2 μm	[78]
	1:1 CL-20/FOX-7	impact and friction sensitivity decreased by 64% and 68%, compared to raw CL-20. the predicted crystal density and detonation parameters are 1.928 g·cm^−3^, 9178 m·s^−1^, and 40.44 GPa	[79]
	ADN/CL-20	*I*_sp_ = 272.6 s	[80]
	HMX/FOX-7	0.1~0.5 μm, impact sensitivity is >45 J, friction sensitivity is 288 N	[81,82]
	1:2 CL-20/3,4-MDNP	density = 1.787 g·cm^–3^, detonation parameters are 8556 m·s^−1^, 31.87 GPa, *E*_50_ is 16 J, friction sensitivity is 180 N	[84]
	2:1 CL-20/3,5-MDNP	density = 1.889 g·cm^–3^, detonation parameters are 9079 m·s^−1^, 37.30 GPa, *E*_50_ is 12 J, friction sensitivity is 120 N	[84]
mixing	NGEC/RDX-GPs	impact sensitivity improved by 15.3%~117.1%, 3.9%~34.6%, 6.9%~31.1% under conditions of -40 °C, 20 °C, and 50 °C; the compression strength improved by 2.5%~23.1%, 10.7%~27.9%, 7.3%~28.5%, the tensile strength was improved by 15.4%~35.0%, 10.4%~33.0%, 11.8%~35.5%	[86]
	HNS/CL-20	density is 1.83 g·cm^–3^, detonation parameters are 8293.19 m·s^−1^, 29.97 GPa,	[87]
	TKX-50/CL-20	*E*_a_ = 185.07 kJ·mol^−1^, impact sensitivity is 4.5 J, friction sensitivity is 216 N	[88]
	FOX-7/HMX	angle of repose is 26.6°,bulk density is 0.522 g·cm^–3^	[17]
	TATB/HMX	CV < 10%, theoretical density is 1.918 g·cm^–3^, detonation parameters are 9027.31 m·s^−1^, 36.34 GPa	[89]
		bulk density is 0.729 g·cm^–3^, real density is 1.925 g·cm^–3^, *H*_50_ is >100 cm	[90]
	CL-20/FOX-7	*D*_50_ = 72.03, 232.16, 322.69 μm	[91]
	TKX-50/CMCAB@CAB	friction sensitivity is 324 N	[92]
	NTO/KC/CTS	*T*_p_ decreased by 14.4 K, *E*_a_ decreased by 290.9 kJ·mol^−1^	[18]
	γ-HMX/GO-TAGP	VoD is 9254 m·s^−1^, impact energy is 26.5 J, friction sensitivity: >360 N	[93]
	TKX-50/TATB	*H*_50_ is 66.1 cm, friction sensitivity is 24%	[94]
	CL-20/Al@Co/NBC	*T*_p_ of Al is 1002.2 °C, impact sensitivity is 30 J, friction sensitivity is 192 N	[98]
	Al@PDA/CL-20	the heat of reaction is 6482 J·g^–1^	[99]
	Al@PDA/HMX	*n* is 0.29 within 1~20 MPa	[100]
	HMX@B/Al/PTFE	initial oxidation temperature of B is 762.45 °C	[101]
	CL-20/Al/F2605	elastic modulus increased by 131%, the detonation speed reached over 8500 m·s^−1^	[102]
	TKX-50/Al/GAP	*E*_a_ increased by 12.07 kJ·mol^−1^, impact and friction sensitivity decreased by 83 J, 80 N	[103]

## Data Availability

Not applicable.
